# The relative importance and interactions of CMR-derived parameters of ventricular mechanics in the prediction of death and transplant late after the Fontan operation

**DOI:** 10.1186/1532-429X-18-S1-O29

**Published:** 2016-01-27

**Authors:** Rahul H Rathod, Lynn A Sleeper, Sunil J Ghelani, Ellen M Keenan, David M Harrild, Andrew J Powell, Tal Geva

**Affiliations:** 1 grid.2515.30000000403788438Cardiology, Boston Children's Hospital, Boston, MA USA; 2 grid.38142.3c000000041936754XPediatrics, Harvard Medical School, Boston, MA USA

## Background

We have previously shown that a larger indexed end-diastolic volume (EDV_i_) of a functional single ventricle as determined by cardiac magnetic resonance (CMR) is an independent predictor of death and heart transplant late after the Fontan operation. Other reports have suggested that decreased ventricular strain and ejection fraction (EF) are associated with poor outcomes. The objective of this study was to identify the relative importance and interactions of CMR-based parameters for risk of death and transplant after the Fontan operation.

## Methods

Clinical CMR studies from 1/2002 to 1/2015 were retrospectively reviewed. Ventricular size and function measurements were calculated using commercially available software (Medis Medical Imaging Systems, Leiden the Netherlands). Global circumferential strain (GCS) and longitudinal strain (GLS) were measured for the single or dominant ventricle at the mid-ventricular level using commercial software (TomTec Imaging Systems, Unterschleissheim, Germany). The primary endpoint was defined as time to death or listing for heart transplantation. Classification and regression tree (CART) survival analysis was performed to identify the subgroups at highest risk for the endpoint without pre-specification of possible interactions. Candidate predictors were indexed EDV, indexed end-systolic volume, EF, indexed ventricular mass, GCS, and GLS. Where applicable, CMR parameters were indexed to BSA^1.3^.

## Results

**Figure 1:**
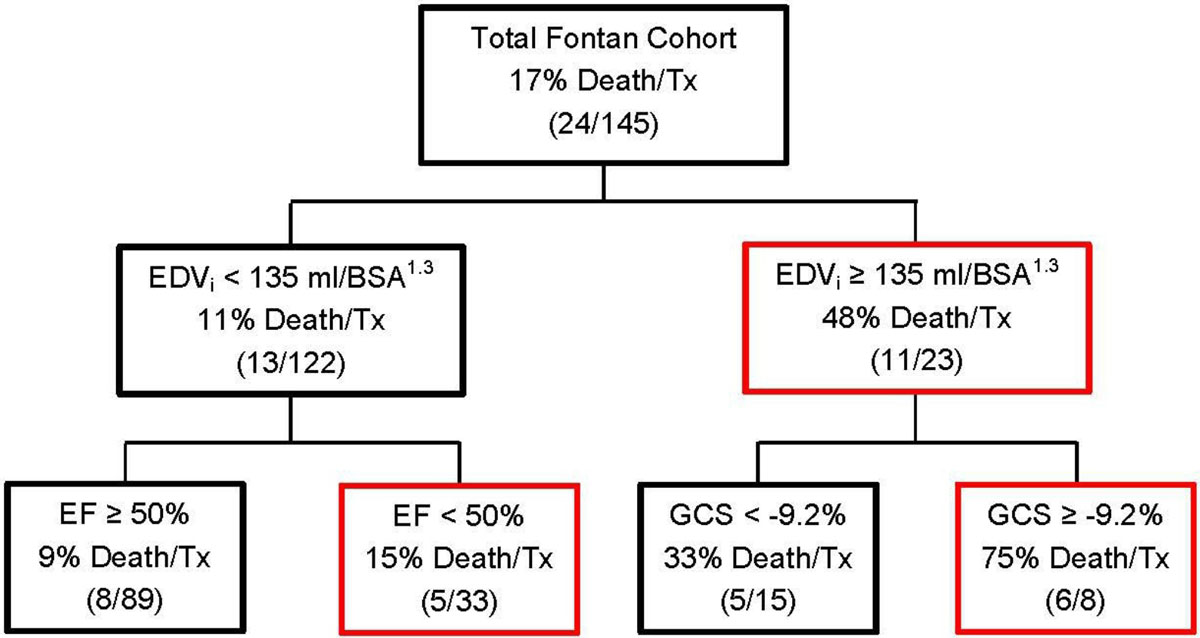
**Classification and regression tree for death and transplant Tx in Fontan paients**.

**Figure 2:**
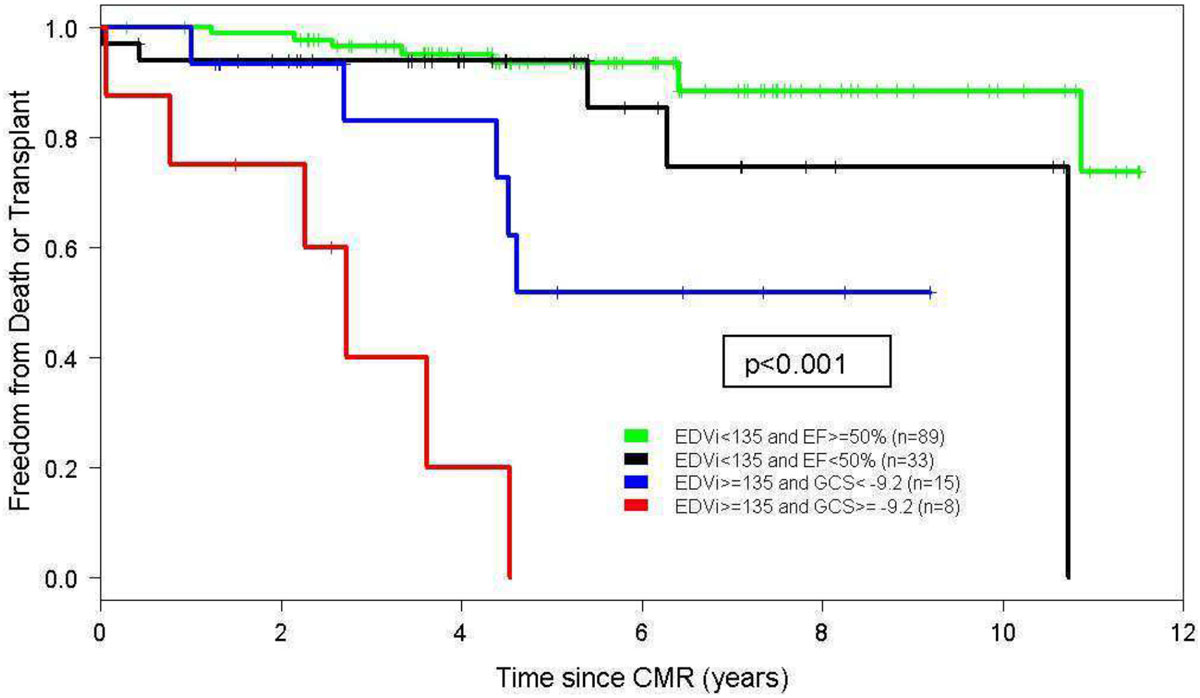
**Freedom from death and transplant by risk factor subgroups**.

The study sample consisted of 145 patients (64% male). Median age at CMR was 16 years [IQR 11-23 years] and age at Fontan was 3.4 years [IQR 2.4-6.2 years]. Over a median follow-up of 4.6 years after CMR, 24 patients (17%) reached the study endpoint (20 deaths, 4 transplant listings). The results of the CART analysis are shown in Figure 1. EDV_i_ was the strongest predictor of transplant-free survival. In the more severely dilated subgroup (EDV_i_ ≥135 mL/BSA^1.3^), worse GCS was the most important predictor of transplant-free survival (33% vs. 75% with endpoint). However, in those with less dilation (EDV_i_ <135 mL/BSA^1.3^), EF <50% was the most important predictor but added little additional discrimination (9% vs. 15% with endpoint). Figure 2 depicts a Kaplan-Meier plot with groups informed by the CART.

## Conclusions

CMR-derived functional single ventricle EDV_i_ is the strongest independent predictor of transplant-free survival in patients late after the Fontan operation. In patients with moderate or worse ventricular dilatation, GCS rather than EF has additional discriminating power for the endpoint. These data highlight the interactions between ventricular dilation and strain and the importance of CMR imaging in this population.

